# Prevalence of unrecognized or “silent” myocardial ischemia in chronic kidney disease patients: Protocol for a systematic review and meta-analysis

**DOI:** 10.1371/journal.pone.0256934

**Published:** 2021-09-02

**Authors:** Christophe Dongmo Fokoua-Maxime, Armel Jackson Seukep, Yahia Bellouche, Takeude Erwan Cheuffa-Karel, Dickson Shey Nsagha, François Folefack Kaze

**Affiliations:** 1 University of New York State—University at Albany School of Public Health, Albany, NY, United States of America; 2 New York State Department of Health, Albany, NY, United States of America; 3 Faculty of Health Sciences, University of Buea, Buea, Cameroon; 4 Brest University Hospital, Brest, France; 5 Faculty of Medicine and Biomedical Sciences, University of Yaoundé I, Yaoundé, Cameroon; 6 Yaoundé University Teaching Hospital, Yaoundé, Cameroon; University of Bologna, ITALY

## Abstract

**Introduction:**

Chronic kidney disease (CKD) patients are at an extremely high risk of silent myocardial ischemia (SMI). However, there is a dearth of evidence on the worldwide prevalence of this very lethal and yet unrecognizable complication of CKD. The proposed systematic review and meta-analysis aims to estimate the global prevalence of SMI among CKD patients.

**Methods and analyses:**

This protocol was conceived according to the preferred reporting items for systematic review and meta-analysis protocols (PRISMA-P) statement. The systematic review will involve all observational studies and clinical trials published until April 30, 2021, and reporting on the prevalence of SMI in CKD patients. Electronic sources including MEDLINE, Embase, Web of Science, and Cochrane database of systematic reviews will be perused for potentially eligible studies, restricted to only studies published in English or French. Two investigators will independently select studies and use a pre-pilot tested form to extract data. Further, they will independently perform a qualitative assessment of the risk of bias and overall quality of the selected studies, followed by a quantitative assessment using funnel plots and Egger’s tests. The heterogeneity between studies will be assessed with the Cochrane’s Q statistic, and the I^2^ statistic will measure the percentage of variation across studies that is due to their heterogeneity rather than chance; the I^2^ will decide if a meta-analysis can be conducted. In case it cannot be conducted, a descriptive analysis will be performed. Otherwise, study-specific estimates will be pooled using either a fixed-effects or a random-effects model, depending on the value of the I^2^ statistic. Subgroup and random effects meta-regression analyses will further investigate the potential sources of heterogeneity. Finally, sensitivity analyses will be performed to measure the impact of low-quality studies on the results of the meta-analysis, and power calculations will determine the probability that we will detect a true effect if it does exist.

**PROSPERO registration number:**

CRD42020211929

**Strengths and limitations of this study:**

The intended systematic review and meta-analysis will fill the knowledge gap on the global prevalence of silent myocardial ischemia (SMI) in CKD patients. The eligible studies will be identified through a methodic literature search followed by a rigorous screening process; we will then use robust meta-analysis tools to pool the data and provide reliable estimates of the global prevalence of SMI in CKD patients. Two major limitations could be: the predominance of clinical trials that might limit the generalizability of the findings, given that some informative patients might have been sidelined by the strict inclusion criteria of these studies; the high probability of type 1 error originating from the important number of subgroup and sensitivity analyses.

## 1-Introduction

Chronic kidney disease (CKD) is a global public health concern [1]. In 2017, the world counted 697.5 million CKD cases and registered 1.2 million deaths attributable to CKD [2]. CKD is an independent risk factor of cardiovascular disease (CVD) [1]. In turn, CVD is the leading cause of morbidity and mortality among patients with CKD [3]. Coronary artery disease (CAD) is responsible for around 50 percent of the deaths of patients with CKD [4–6]. Research has highlighted the uniqueness of CAD in patients with CKD—as compared with the general population—because of an early onset, a more rapid progression and atypical symptoms [7]. CKD patients are at a high risk of silent myocardial ischemia (SMI) because like diabetes, CKD is associated with neuronal disturbances [8] that can alter the patients’ perception of pain. Another hypothesis proposes that SMI among CKD patients is due to the CKD-induced microinflammatory state [9] which disrupts the balance between proinflammatory and anti-inflammatory cytokines that is needed for the perception of chest pain [10]. Finally, SMI is well-documented in dialysis [11–14]; indeed, the hemodialysis procedure itself provokes regional wall motion abnormalities and disruptions in the myocardial perfusion [15]. Thus, CKD is a major risk factor for SMI.

In 2017, the number of deaths due to CKD plus the number of deaths of CVD patients imputable to impaired kidney function represented 4·6% of the total death toll in the world [2]. Many of these deaths were probably induced by silent myocardial ischemia that went unnoticed and so were not diagnosed, consequently, they did not receive proper care and ultimately resulted in earlier deaths. Therefore, it is urgent to take appropriate preventive measures to quell the deleterious effects of this highly lethal and imperceptible complication of CKD. A key pre-requisite for this task is to know the proportion of CKD patients in the world who suffer from SMI, as well as the potential factors which contribute to the late or non-diagnosis of SMI in CKD patients. To date, no study has attempted to provide such information. The proposed systematic review and meta-analysis aims to fill this gap in the literature by providing a reliable estimate of the global prevalence of SMI among patients with CKD.

## 2-Objective

To determine the global prevalence of silent or unrecognized myocardial ischemia (SMI) among patients with CKD.

## 3-Review question

What is the prevalence of silent or unrecognized myocardial ischemia among patients with CKD?

## 4-Methods

The present protocol has been drafted in line with the preferred reporting items for systematic review and meta-analysis protocols (PRISMA-P) statement [16]. This protocol will be registered in the international prospective register of systematic reviews (PROSPERO) network. The anticipated systematic review and meta-analysis will agree with this protocol, and will be reported based on the meta-analyses of observational studies (MOOSE) guidelines [17] and the Preferred Reporting Items for Systematic reviews and Meta-Analysis (PRISMA) guidelines [18].

### 4.1-Search methods for the identification of eligible studies

A trained and experienced medical librarian will perform a systematic and comprehensive search in the following databases from inception until July 31, 2021: MEDLINE/ PubMed (1947 toJuly 31, 2021), Embase (1973 to July 31, 2021), Web of Science (1985 to July 31, 2021) and Cochrane Central Register of Controlled Trials (1991 to July 31, 2021). Key terms that will be included in the search are: “silent myocardial ischemia”, “silent ischemic heart disease”, “silent coronary artery disease”, “silent coronary heart disease”, “chronic kidney disease”, “chronic renal disease”, “chronic kidney failure”, “chronic renal failure”, “chronic kidney disorder”, “chronic renal disorder”. The search strategy for MEDLINE (via PubMed) is presented in the S1 Appendix. Following the search in the databases, we will peruse the reference lists of all selected articles to identify potential supplementary data sources.

The search will be repeated prior to the publication of the systematic review in order to include any potential eligible study that could have been published since the end of the initial electronic search.

### 4.2-Eligibility criteria for study selection

#### 4.2.1-Study design

The review will include all cross-sectional, cohort, case-control studies and clinical trials published until April 30, 2021. The search will be supplemented by hand searches in the reference lists of included studies and systematic searches in the gray literature for additional relevant articles.

#### 4.2.2-Participants

Study subjects in the eligible studies must be individuals diagnosed with any stage of CKD. There will be no restrictions based on sex, age, race/ethnicity, socioeconomic status, history of previous myocardial infarction, or geographic region.

#### 4.2.3-Clinical outcomes

CKD described in the eligible studies must have been identified by a physician and the diagnosis must have been made based on the criteria from the Improving Global Outcomes (KDIGO) Consensus Conference which defines CKD as kidney damage (albumin-to-creatinine ratio > 30 mg/g in two of three spot urine specimens) or glomerular filtration rate (GFR) < 60 mL/min/1.73m^2^ for 3 months or more, irrespective of the cause [19–21]. Silent myocardial ischemia (SMI) must have been diagnosed by a physician and diagnosed in patients who display no symptoms during an exercise or pharmaceutical stress test but who exhibit transient ST-segment changes, perfusion defects, or reversible regional wall motion abnormalities [22], or patients who display no symptoms and a myocardial ischemia is later diagnosed based on myocardial imaging evidence or pathological findings on autopsy [23, 24].

#### 4.2.4-Outcome measure

The outcome of focus of this review will be the prevalence of SMI in CKD patients.

#### 4.2.5-Language

The search will be restricted to only studies published in English or French.

#### 4.2.6-Exclusion criteria

We will exclude book chapters, (systematic) reviews and meta-analyses, case-series, fact sheets, white papers, conference proceedings, letters to the editor, commentaries, editorials, and studies without primary data and/or with unachieved methods description. For search leading to similar publications (duplicates), only the most comprehensive report including the largest sample size, a complete methods section, and an entire results report will be included. Also, *Kin relationships*, defined as multiple publications describing the same or overlapping series of patients, will be identified; in this instance, only the study with the largest sample size, a comprehensive methods description, and a complete results section will be selected.

The complete bibliography of the validated and included as well as the rejected studies will be available by request to the corresponding author.

### 4.3-Data collection and analysis

#### 4.3.1-Data compilation

Search results will be imported into EndNote X9.3.3 (Clarivate Analytics, Philadelphia, USA). Duplicate articles will be removed, and the remaining references will be alphabetically ordered according to the first authors’ names.

#### 4.3.2-Selection of studies

**Fig 1:**
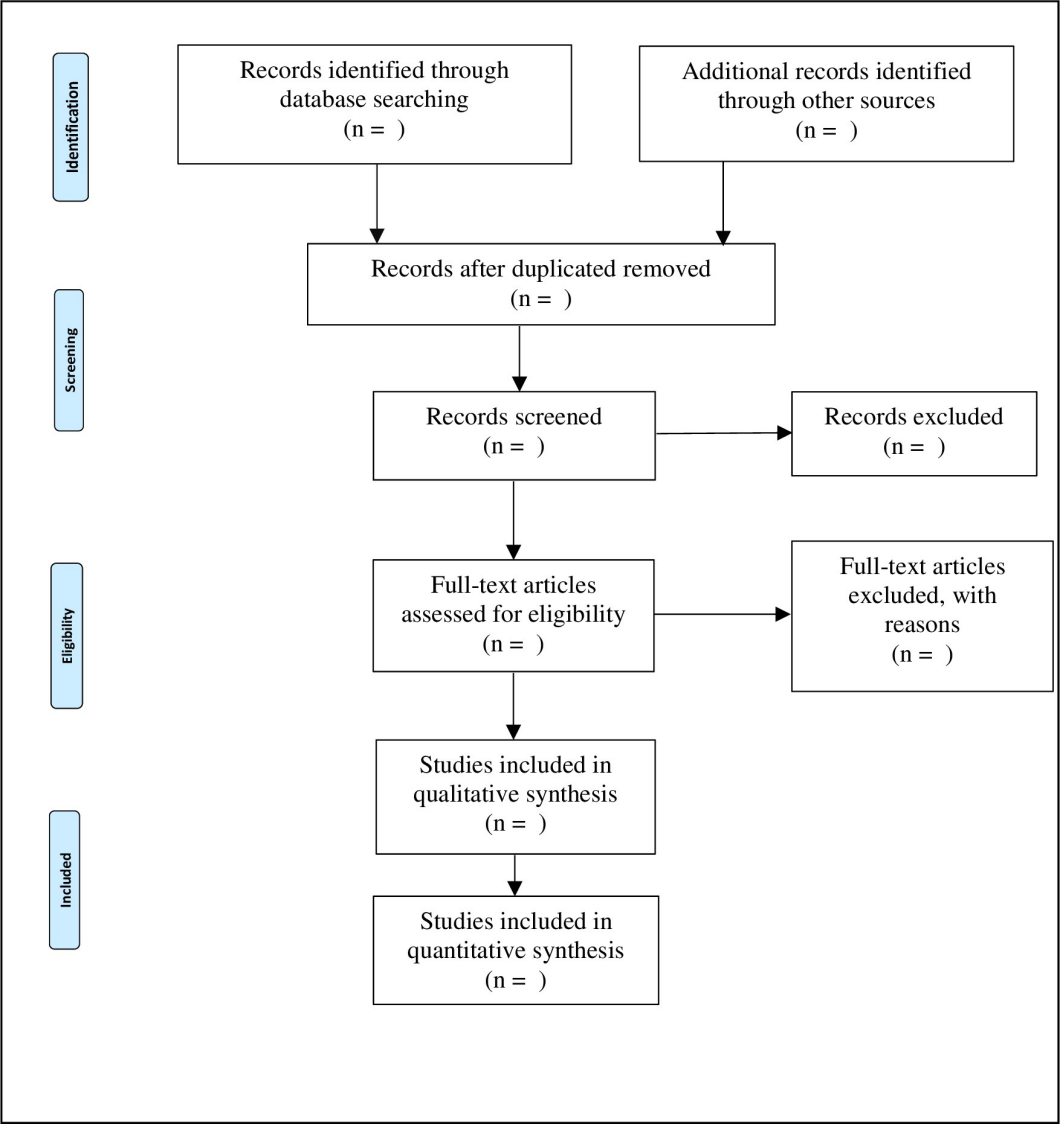
PRISMA flow diagram of the selection of studies to include in a systematic review.

Two authors (DCFM, AJS) will independently screen titles and abstract records that were imported after the literature search. Consecutive to the initial screening, the full texts of records considered eligible will be retrieved and further evaluated for inclusion by the same researchers. Discrepancies in the list of selected articles will be resolved by consensus or by a third reviewer (YB) if necessary. In line with the PRISMA guidelines [16], a flow diagram will summarize the entire study selection process (Fig 1). The anticipated start date for the selection of articles is August 1, 2021 and the expected completion date is August 31, 2021.

#### 4.3.3- Data extraction and management

Two authors (DCFM, AJS) will use a pre-pilot tested and systematized data extraction form to collect data on:

- Study identification: first author’s name, year of publication, country and/or region
- Study characteristics: study design (cross-sectional, cohort, case-control study, or randomized control trial), setting (hospital- or community-based), duration of follow-up for cohort studies and clinical trials, number of controls per cases, and type of matching (if present) for case-control studies.
- Study population: sample size, mean or median age, age range, sex ratio, race/ethnicity distribution.
- Primary exposure: stage of CKD
- Primary outcome: SMI
- Covariates: glomerular filtration rate, creatinine, albuminuria, urine albumin-to-creatinine ratio, type of treatment received (medicine, dialysis, or both), method used to detect the SMI (ECG, medical imaging, or autopsy), duration since the diagnosis of CKD, mean or median age at diagnosis of CKD, proportion of patients with a history of 1 or more acute complications of CKD (edema, gout, metabolic acidosis, hyperkalemia), proportion of patients with 1 or more chronic complications of CKD (anemia, renal osteodystrophy, left ventricular hypertrophy, dyslipidemia, malnutrition), body mass index (BMI), proportion of patients smoking, proportion of patients with diabetes, proportion of patients with hypertension, proportion of patients with other cardiovascular diseases (heart failure, arrhythmias, heart valve disease, cardiomyopathies, pericarditis, aorta diseases, stroke), proportion of patients with 1 or more other major comorbidities (HIV/AIDS, cancer, chronic obstructive pulmonary disease).
- Epidemiological measure: prevalence.

We will contact the corresponding author(s) of articles from which we could not extract important information during the retrieval process.

Finally, two other authors (YB, TECK) will randomly select 5 studies each and will extract the data one more time to validate that the data extraction process was performed accurately.

#### 4.3.4-Quality assessment and risk of bias of selected studies

Two authors (DCFM, ELY) will separately appraise the methodological quality of each included study, using the tool conceived by Hoy et al for prevalence studies [25]. Each item will be assigned a score of 1 (yes) or 0 (no), and scores will be summed across items to generate an overall quality score that will range from 0 to 10. According to the overall scores, each of the two authors (DCFM, AJS) will classify studies as having a low (>8), moderate (6–8), or high (≤5) risk of bias. Further, the non-weighted Cohen’s kappa statistic will be used to assess the level of agreement between the reviewers. In case of substantial disagreement between the two reviewers (DCFM, AJS), a third reviewer (YB) will be solicited for arbitration. Following this evaluation, authors of publications containing confusing results and/or results prone to multiple interpretations will be reached out by email to request some clarification or supplemental information. In case a study is excluded, the reasons will be explicitly presented.

If more than 10 eligible studies are found, then publication bias will be evaluated by the symmetry of funnel plots, supplemented by a quantitative analysis through an Egger’s test.

#### 4.3.5-Data synthesis

The data of the included studies will be summarized in ad hoc tables. The heterogeneity between studies will be assessed with the Cochrane’s Q statistic. Further, the *I*^*2*^ statistic will be used to measure the percentage of variation across studies that is due to their heterogeneity rather than chance [19, 20]. The value of the *I*^2^ statistic will be classified as small if 0 ≤ *I*^2^ ≤ 25%, medium if 25% < *I*^2^ ≤ 50%, and large if *I*^2^ > 50% [26, 27]. The category of the *I*^*2*^ statistic will determine whether a meta-analysis is possible. If the *I*^2^ statistic is large, then a meta-analysis will be considered not possible, and a descriptive analysis will be performed. Otherwise, a meta-analysis will be deemed feasible, and the category of the *I*^2^ statistic will further decide the type of statistical model to be used to pool the study-specific estimates. If the I^2^ statistic is small then a fixed-effects model analysis will be conducted, otherwise, a random-effect model analysis [28] will be performed, after stabilizing the variance of individual studies with the Freeman-Tukey double arc-sine transformation [29]. R software version 3.6.1 (R Core Team, Vienna, Austria) will be used to combine data, along with 95% confidence intervals.

#### 4.3.6. Sources of heterogeneity

The potential sources of heterogeneity will be investigated by subgroup and meta-regression analyses [26]. Subgroup analyses will be performed by stage of CKD, dialysis status, sex, menopause status, race, obesity status, diabetes status, and hypertension status. If more than 10 studies are included in the quantitative synthesis, then subgroup analyses will be supplemented by random effect meta-regression analyses which will allow the effects of multiple factors (called effect modifiers) to be simultaneously investigated [30]. The potential effect modifiers considered will be the following: sex, race, obesity status, diabetes status, hypertension status, and type of study (observational vs experimental). We will use the model F value and its statistical significance to assess whether there is evidence for an association between any of the covariates and the outcome; all the covariates with p-value <0.2 in bivariate models will be added to the multivariable model, in which a p-value <0.05 will be considered statistically significant. The model fit will be assessed using the proportion of the between-study variance explained by the covariates (adjusted R^2^) [31]. To control for the risk of type I error when performing meta-regression with multiple covariates, we will perform Monte Carlo permutation tests to calculate P values adjusted for type 1 error and we will check if there is a change in statistical significance [31, 32].

#### 4.3.7. Robustness of the results

The robustness of the results will be assessed by performing sensitivity analyses to measure the impact of low-quality studies (identified through their risk of bias). Low-quality studies will be removed one at a time and the meta-analysis will be performed again; we will then compare the results of the meta-analysis with and without the study being assessed, while also accounting for the study sample size, strength of evidence, and impact on aggregated effect size. However, if all the selected studies are at a high risk of bias, we will not conduct sensitivity analyses.

#### 4.3.8. Power analyses

Power analyses will measure the probability that we will detect a true effect if it does exist. Assuming a normal distribution of the effect estimates, the power will be:

Power=1–β[33] (1)

$$\text{with}\;\beta =Ф\;\left({\text{C}}_{\alpha }-\lambda \right)-Ф\;\left(-{\text{C}}_{\alpha }-\lambda \right),$$ (2)

where β is the probability of type II error or false negative rate,
C_α_ represents the critical value of a *Z*-distribution,
Φ is the standard normal density function obtained through the formula $\Phi =\frac{1}{\sqrt{2\pi }}e{-}^{Z^{2}/2}$,
λ is the true value defined as $\lambda =\frac{1}{\sqrt{Vϙ}}$, with ϙ being the true effect size and *V*_*ϙ*_ its variance.

Under the assumption that the heterogeneity between the selected studies will be moderate, *V*_*ϙ*_ will be calculated according to the method of Hedges and Pigott: *V*_*ϙ*_ = 1.67 x Vy/*k* [34], with *k* being the number of included studies and Vy being the between-study variance.

The power calculations will be performed with the *power*.*analysis* function contained in the *dmetar* package of the statistical software R version 4.0.4. Under the aforementioned assumptions, if the true effect is 0.4 and 10 studies are included in the final analyses, then our study will have an 85% power.

#### 4.3.9. Ethics and dissemination

This systematic review was considered exempt from Institutional Review Board approval since our analyses will only include previously published non-identifiable data. The anticipated systematic review and meta-analysis will be reported following the meta-analyses of observational studies (MOOSE) guidelines and the Preferred Reporting Items for Systematic Reviews and Meta-Analyses (PRISMA) statement. The results of the systematic review and meta-analysis will be made available through conference proceedings and peer-reviewed publications.

## 5- Strengths and limitations of this study

The intended systematic review and meta-analysis will fill the knowledge gap on the global prevalence of silent myocardial ischemia (SMI) in CKD patients. The eligible studies will be identified through a methodic literature search followed by a rigorous screening process; we will then use robust meta-analysis tools to pool the data and provide reliable estimates of the global prevalence of SMI in CKD patients. Two major limitations could be: the predominance of clinical trials that might limit the generalizability of the findings, given that some informative patients might have been sidelined by the strict inclusion criteria of these studies; the high probability of type 1 error originating from the important number of subgroup and sensitivity analyses.

## 6-Conclusion

CKD is a major contributor to the mortality and morbidity in the world. Coronary artery disease (CAD) is responsible for around half of the deaths of CKD patients, yet many of these cases are silent or unrecognized because of the biochemical and morphological changes that accompany CKD; thus, these cases are not medically attended and subsequently lead to earlier deaths. Therefore, it is urgent to take appropriate preventive measures to quell the deleterious effects of this highly lethal and imperceptible complication of CKD. However, there is a gap in our knowledge about the worldwide prevalence and risk factors of silent myocardial ischemia (SMI) in CKD patients. This systematic review will fill this gap by estimating the pooled global prevalence of SMI in CKD patients. It will provide key evidence that will help the worldwide scientific community in its efforts to quell the deleterious effects of this highly lethal and yet unrecognizable complication of CKD.

## Supporting information

S1 Checklist PRISMA-P 2015 checklist. (DOCX)

S1 Appendix MEDLINE(PubMed) search strategy. (DOCX)
